# Crystal structure of chlorido­(piperidine-κ*N*)(quinoline-2-carboxyl­ato-κ^2^ *N*,*O*)platinum(II)

**DOI:** 10.1107/S160053681401191X

**Published:** 2014-06-23

**Authors:** Chi Nguyen Thi Thanh, Ngan Nguyen Bich, Luc Van Meervelt

**Affiliations:** a Chemistry Department, Hanoi National University of Education, 136 – Xuan Thuy – Cau Giay, Hanoi, Vietnam; b Chemistry Department, KU Leuven, Celestijnenlaan 200F, B-3001 Leuven (Heverlee), Belgium

**Keywords:** crystal structure, *cis*-platinum(II) complexes, hydrogen bonding, anti­cancer activity

## Abstract

The title compound, [Pt(C_10_H_6_NO_2_)Cl(C_5_H_11_N)], crystallizes with one mol­ecule in the asymmetric unit. The Pt^II^ cation has a slightly distorted square-planar coordination environment defined by a chloride anion, the quinoline N atom and a carboxyl­ate O atom of the bidentate quinaldate ligand and a piperidine N atom. An intra­molecular C—H⋯Cl hydrogen bond occurs. In the crystal, mol­ecules are stacked into columns along the *c* axis by the formation of N—H⋯Cl and C—H⋯O hydrogen bonds.

## Abstract 2

The platinum(II) complex with notable antitumor activity shows a slightly distorted square-planar coordination and intramolecular C—H⋯Cl and intermolecular N—H⋯Cl and C—H⋯O hydrogen bonds.

## Chemical context   

The title compound belongs to a series of platinum(II) complexes bearing piperidine (pip) as a ligand, which exhibit notable anti­tumour activity (Da *et al.*, 2001 ▶; Rounaq Ali Khan *et al.*, 2000 ▶; Solin *et al.*, 1982 ▶). In comparison with the earlier reported complex [PtCl_2_(pip)(quinoline)] (Nguyen Thi Thanh *et al.*, 2014 ▶), the quinoline ligand is replaced by an *N*,*O*-bidentate quinaldate ligand. It is inter­esting to note that in the [PtCl_2_(pip)(quinoline)] complex, the quinoline and piperidine ligands are arranged in *cis* positions (Nguyen Thi Thanh *et al.*, 2014 ▶). In the title compound, the quinoline ring of the quinaldate ligand occupies a *trans* position with respect to the piperidine ring. We suggest that in the reaction solution there exists a chemical equilibrium between the neutral and bipolar forms of quinaldic acid. Thus, the quinaldic acid in its ionic form coordinates with Pt^II^ *via* the O atom of the carboxyl­ate group first and in a *cis* position with respect to piperidine based on the *trans* effect. In a second step, the quinaldic acid coordin­ates with Pt^II^ also *via* its N atom, resulting in the cyclic complex.
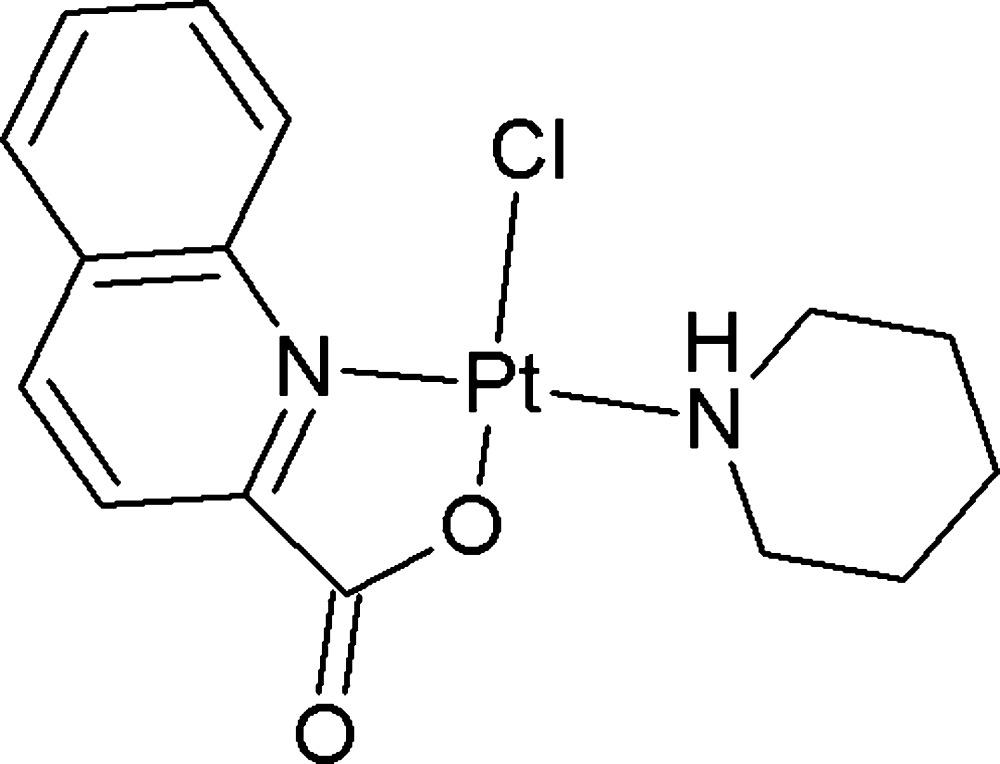


The anti­cancer activity of the title compound was tested according to the method described in Skehan *et al.* (1990 ▶) on four human cancer cells of HepG2, RD, MCF7 and Fl. The IC_50_ values calculated based on OD values taken on an Elisa instrument at 515–540 nm are 4.46, 2.59, >10 and 5.60 µg ml^−1^, respectively.

## Structural commentary   

**Figure 1:**
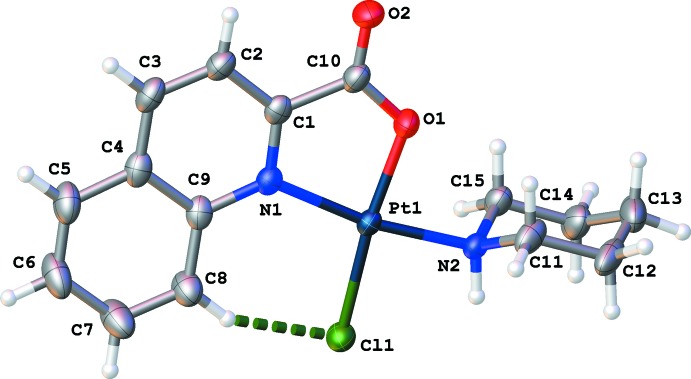
View of the mol­ecular structure of the title compound, showing the atom-labelling scheme, with ellipsoids drawn at the 50% probability level. The intra­molecular C—H⋯Cl hydrogen bond is shown as a green dashed line.

The title complex crystallizes with one mol­ecule per asymmetric unit (Fig. 1 ▶). The Pt^II^ cation is surrounded by two N atoms, one O atom and one Cl atom, resulting in a slightly distorted square-planar coordination environment [angles around platinum: O1—Pt1—N1 81.38 (9), O1—Pt1—N2 88.26 (9), Cl1—Pt1—N2 84.26 (7) and Cl1—Pt1—N1 106.11 (7)°]. The Cl^−^ and the Pt^II^ atoms are displaced from the least-squares plane of the quinoline ring and all other coord­inating atoms by 0.2936 (7) and 0.0052 (1) Å, respectively. The piperidine ring adopts a chair conformation and is almost perpendicular to the coordination plane of the Pt^II^ cation [dihedral angle between the best plane through the piperidine ring and the four atoms coordinating to the Pt^II^ cation = 79.66 (13)°]. Bond lengths are normal and agree well with related platinum compounds (Cambridge Structural Database, version 5.34; Allen, 2002 ▶). There is an intra­molecular hydrogen bond between atom Cl1 and atom H8 (Fig. 1 ▶ and Table 1 ▶).

## Supra­molecular features   

**Figure 2:**
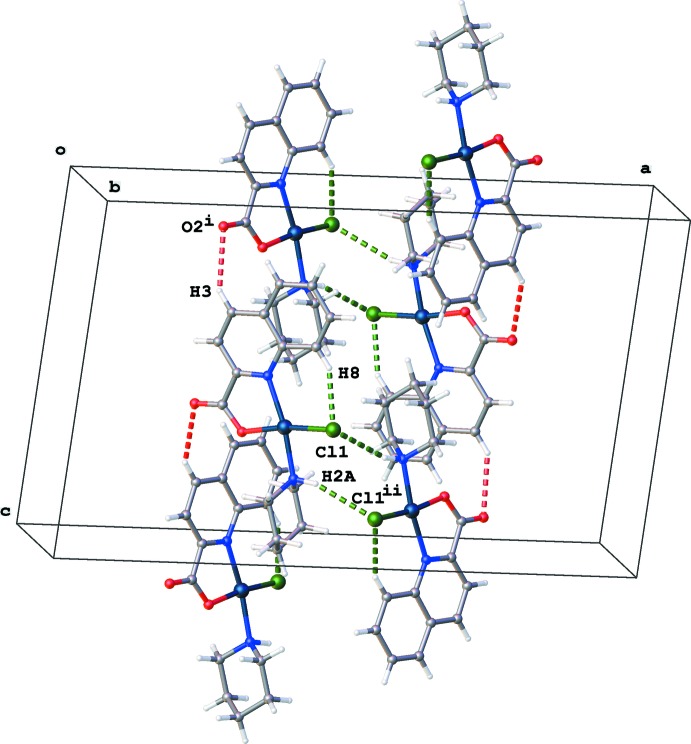
View of the crystal packing for the title compound, with (N/C)—H⋯Cl and C—H⋯O hydrogen bonds drawn as green and red dashed lines, respectively. [Symmetry codes: (i) *x*, −*y* + 1, *z* − 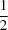; (ii) −*x* + 1, *y*, −*z* + 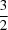.]

The crystal packing is characterized by N—H⋯Cl and C—H⋯O hydrogen bonds (Table 1 ▶). Mol­ecules are arranged into columns along the *c* axis (Fig. 2 ▶) with the piperidine rings all directed towards the center of the column, favouring hydro­phobic inter­actions.

## Synthesis and crystallization   

The starting complex K[PtCl_3_(piperidine)] (0.425 g, 1 mmol), prepared according to the synthetic protocol of Da *et al.* (2001 ▶), was dissolved in water (10 ml) and filtered to afford a clear solution. To this solution, quinaldic acid (1.2 mmol) in an aqueous ethanol solution (5 ml, 1:1 *v*/*v*) was added gradually while stirring at room temperature for 1 h. The reaction mixture was stirred further for 4 h. The precipitated yellow substance was filtered off and washed consecutively with a 0.1 *M* HCl solution (2 × 2 ml), warm water (3 × 2 ml) and cold ethanol (2 ml). The product was then dried in a vacuum at 323 K for 4 h. The yield was 80%. Single crystals suitable for X-ray diffraction analysis were obtained by slow evaporation from an ethanol–water (1:1 *v*/*v*) solution at room temperature. Positive ESI–MS: *m*/*z* 1973 [4*M* + Na]^+^, 1483 [3*M* + Na]^+^, 998 [2*M* + Na]^+^, 510 [*M* + Na]^+^, 977 [2*M* + H]^+^, 489 [*M* + H]^+^; IR (KBr) cm^−1^: 3192 (ν_NH_); 3080, 2930, 2866 (ν_CH_); 1678 (ν_C=O_); 1592, 1459 (ν_C=C arom_); 1334 (ν_C—O_); ^1^H NMR (δ p.p.m; CDCl_3_, 500Hz): 9.50 (1H, *d*, ^3^ *J* = 9.0 Hz, Ar-*H*), 8.51 (1H, *d*, ^3^ *J* = 8.0 Hz, Ar-*H*), 8.06 (1H, *d*, ^3^ *J* = 8.0 Hz, Ar-*H*), 7.91–7.88 (2H, *ov*, Ar-*H*), 7.71 (1H, *t*, ^3^ *J* = 8.0 Hz, Ar-*H*), 3.52 (2H_α_^e^, *d*, ^2^ *J*_ae_ = 12.5 Hz, C_5_ *H_10_*NH), 3.27 (2H_α_^a^, *q*, ^2^ *J*_ae_, ^3^ *J*_aa_, ^3^ *J*_aa(NH)_ = 12.5 Hz, C_5_ *H_10_*NH), 1.76–1.61 (4H_β_, 2H_γ_, *ov*, C_5_ *H_10_*NH), 4.00 (1H, *br*, C_5_H_10_N*H*).

## Refinement   

All H atoms were refined using a riding model, with C—H = 0.95 Å for aromatic, C—H = 0.99 Å for CH_2_ and N—H = 0.93 Å for amino H atoms, with *U*_iso_ = 1.2*U*_eq_(C,N).

## Supplementary Material

Crystal structure: contains datablock(s) I. DOI: 10.1107/S160053681401191X/wm0005sup1.cif

Structure factors: contains datablock(s) I. DOI: 10.1107/S160053681401191X/wm0005Isup2.hkl

CCDC reference: 1004305

Additional supporting information:  crystallographic information; 3D view; checkCIF report

## Figures and Tables

**Table 1:** Hydrogen-bond geometry (Å, °)

*D*—H⋯*A*	*D*—H	H⋯*A*	*D*⋯*A*	*D*—H⋯*A*
N2—H2*A*⋯Cl1^i^	0.93	2.74	3.624 (2)	160
C3—H3⋯O2^ii^	0.96	2.53	3.360 (4)	145
C8—H8⋯Cl1	0.95	2.40	3.268 (3)	152

Symmetry codes: (i) 
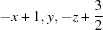
; (ii) 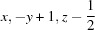.

**Table 2:** Experimental details

Crystal data	
Chemical formula	[Pt(C_10_H_6_NO_2_)Cl(C_5_H_11_N)]
*M*_r_	487.85
Crystal system, space group	Monoclinic, *C*2/*c*
Temperature (K)	200
*a*, *b*, *c* (Å)	22.7542 (8), 9.7540 (3), 14.0139 (5)
β (°)	95.542 (3)
*V* (Å^3^)	3095.78 (19)
*Z*	8
Radiation type	Mo *K*α
μ (mm^−1^)	9.24
Crystal size (mm)	0.3 × 0.3 × 0.2

Data collection	
Diffractometer	Agilent SuperNova (single source at offset, Eos detector)
Absorption correction	Multi-scan (*CrysAlis PRO*; Agilent, 2012 ▶)
*T*_min_, *T*_max_	0.473, 1.000
No. of measured, independent and observed [*I* > 2σ(*I*)] reflections	31419, 3166, 2951
*R*_int_	0.026
(sin θ/λ)_max_ (Å^−1^)	0.625

Refinement	
*R*[*F* ^2^ > 2σ(*F* ^2^)], *wR*(*F* ^2^), *S*	0.015, 0.035, 1.12
No. of reflections	3166
No. of parameters	190
H-atom treatment	H-atom parameters constrained
Δρ_max_, Δρ_min_ (e Å^−3^)	0.80, −0.53

Computer programs: *CrysAlis PRO* (Agilent, 2012 ▶), *SHELXS97* and *SHELXL97* (Sheldrick, 2008 ▶) and *OLEX2* (Dolomanov *et al.*, 2009 ▶).

## supplementary crystallographic information

### Crystal data

**Table:**

[Pt(C_10_H_6_NO_2_)Cl(C_5_H_11_N)]	*F*(000) = 1856
*M**_r_* = 487.85	*D*_x_ = 2.093 Mg m^−^^3^
Monoclinic, *C*2/*c*	Mo *K*α radiation, λ = 0.7107 Å
*a* = 22.7542 (8) Å	Cell parameters from 16449 reflections
*b* = 9.7540 (3) Å	θ = 3.4–29.8°
*c* = 14.0139 (5) Å	µ = 9.24 mm^−^^1^
β = 95.542 (3)°	*T* = 200 K
*V* = 3095.78 (19) Å^3^	Block, yellow
*Z* = 8	0.3 × 0.3 × 0.2 mm

### Data collection

**Table:**

Agilent SuperNova (single source at offset, Eos detector) diffractometer	3166 independent reflections
Radiation source: SuperNova (Mo) X-ray Source	2951 reflections with *I* > 2σ(*I*)
Mirror monochromator	*R*_int_ = 0.026
Detector resolution: 15.9631 pixels mm^-1^	θ_max_ = 26.4°, θ_min_ = 2.8°
ω scans	*h* = −28→28
Absorption correction: multi-scan (*CrysAlis PRO*; Agilent, 2012)	*k* = −12→12
*T*_min_ = 0.473, *T*_max_ = 1.000	*l* = −17→17
31419 measured reflections	

### Refinement

**Table:**

Refinement on *F*^2^	Primary atom site location: structure-invariant direct methods
Least-squares matrix: full	Secondary atom site location: difference Fourier map
*R*[*F*^2^ > 2σ(*F*^2^)] = 0.015	Hydrogen site location: inferred from neighbouring sites
*wR*(*F*^2^) = 0.035	H-atom parameters constrained
*S* = 1.12	*w* = 1/[σ^2^(*F*_o_^2^) + (0.0118*P*)^2^ + 9.138*P*] where *P* = (*F*_o_^2^ + 2*F*_c_^2^)/3
3166 reflections	(Δ/σ)_max_ = 0.002
190 parameters	Δρ_max_ = 0.80 e Å^−^^3^
0 restraints	Δρ_min_ = −0.53 e Å^−^^3^

### Special details

**Table:**

Geometry. All e.s.d.'s (except the e.s.d. in the dihedral angle between two l.s. planes) are estimated using the full covariance matrix. The cell e.s.d.'s are taken into account individually in the estimation of e.s.d.'s in distances, angles and torsion angles; correlations between e.s.d.'s in cell parameters are only used when they are defined by crystal symmetry. An approximate (isotropic) treatment of cell e.s.d.'s is used for estimating e.s.d.'s involving l.s. planes.
Refinement. Refinement of *F*^2^ against ALL reflections. The weighted *R*-factor *wR* and goodness of fit *S* are based on *F*^2^, conventional *R*-factors *R* are based on *F*, with *F* set to zero for negative *F*^2^. The threshold expression of *F*^2^ > σ(*F*^2^) is used only for calculating *R*-factors(gt) *etc*. and is not relevant to the choice of reflections for refinement. *R*-factors based on *F*^2^ are statistically about twice as large as those based on *F*, and *R*- factors based on ALL data will be even larger.

### Fractional atomic coordinates and isotropic or equivalent isotropic displacement parameters (Å^2^)

**Table:**

	*x*	*y*	*z*	*U*_iso_*/*U*_eq_	
C1	0.29348 (12)	0.6098 (3)	0.5028 (2)	0.0268 (6)	
C2	0.25609 (13)	0.5746 (3)	0.4211 (2)	0.0327 (7)	
H2	0.2227	0.5176	0.4264	0.039*	
C3	0.26831 (14)	0.6232 (3)	0.3338 (2)	0.0354 (7)	
H3	0.2432	0.6014	0.2777	0.042*	
C4	0.31811 (14)	0.7052 (3)	0.3278 (2)	0.0324 (7)	
C5	0.33360 (18)	0.7554 (4)	0.2386 (2)	0.0432 (8)	
H5	0.3085	0.7372	0.1818	0.052*	
C6	0.38367 (19)	0.8290 (4)	0.2332 (2)	0.0516 (10)	
H6	0.3934	0.8625	0.1730	0.062*	
C7	0.42105 (18)	0.8556 (4)	0.3167 (2)	0.0503 (9)	
H7	0.4565	0.9057	0.3121	0.060*	
C8	0.40769 (16)	0.8111 (3)	0.4047 (2)	0.0400 (8)	
H8	0.4336	0.8309	0.4603	0.048*	
C9	0.35561 (14)	0.7362 (3)	0.4130 (2)	0.0285 (6)	
C10	0.28109 (13)	0.5514 (3)	0.5984 (2)	0.0318 (7)	
C11	0.42217 (15)	0.5840 (3)	0.8206 (2)	0.0402 (8)	
H11A	0.4518	0.5401	0.7832	0.048*	
H11B	0.3857	0.5281	0.8125	0.048*	
C12	0.44551 (16)	0.5867 (4)	0.9262 (3)	0.0479 (9)	
H12A	0.4509	0.4914	0.9499	0.058*	
H12B	0.4845	0.6323	0.9333	0.058*	
C13	0.40413 (18)	0.6613 (5)	0.9861 (2)	0.0550 (10)	
H13A	0.4225	0.6690	1.0528	0.066*	
H13B	0.3671	0.6083	0.9869	0.066*	
C14	0.39015 (16)	0.8039 (4)	0.9459 (2)	0.0443 (9)	
H14A	0.4263	0.8607	0.9527	0.053*	
H14B	0.3604	0.8481	0.9828	0.053*	
C15	0.36650 (13)	0.7956 (3)	0.8404 (2)	0.0337 (7)	
H15A	0.3285	0.7455	0.8344	0.040*	
H15B	0.3591	0.8893	0.8150	0.040*	
Cl1	0.45271 (3)	0.88368 (8)	0.62745 (5)	0.03279 (16)	
N1	0.34113 (10)	0.6894 (2)	0.50082 (16)	0.0255 (5)	
N2	0.40915 (10)	0.7240 (2)	0.78242 (16)	0.0264 (5)	
H2A	0.4443	0.7733	0.7893	0.032*	
O1	0.31888 (9)	0.5856 (2)	0.66939 (14)	0.0369 (5)	
O2	0.23904 (10)	0.4760 (3)	0.60452 (16)	0.0433 (6)	
Pt1	0.380802 (4)	0.720087 (11)	0.639573 (7)	0.02386 (4)	

### Atomic displacement parameters (Å^2^)

**Table:**

	*U*^11^	*U*^22^	*U*^33^	*U*^12^	*U*^13^	*U*^23^
C1	0.0264 (14)	0.0266 (14)	0.0271 (14)	0.0071 (12)	0.0005 (11)	−0.0021 (12)
C2	0.0288 (15)	0.0374 (17)	0.0308 (15)	0.0016 (13)	−0.0034 (12)	−0.0040 (13)
C3	0.0374 (16)	0.0387 (18)	0.0281 (15)	0.0067 (14)	−0.0071 (12)	−0.0063 (13)
C4	0.0425 (17)	0.0282 (16)	0.0256 (15)	0.0079 (13)	−0.0006 (12)	−0.0035 (12)
C5	0.067 (2)	0.0370 (18)	0.0247 (16)	−0.0022 (17)	−0.0017 (15)	−0.0011 (13)
C6	0.086 (3)	0.042 (2)	0.0269 (16)	−0.014 (2)	0.0094 (17)	0.0007 (15)
C7	0.071 (3)	0.046 (2)	0.0345 (18)	−0.0231 (19)	0.0125 (17)	−0.0054 (16)
C8	0.052 (2)	0.0383 (18)	0.0298 (16)	−0.0106 (15)	0.0033 (14)	−0.0039 (13)
C9	0.0397 (16)	0.0213 (14)	0.0242 (14)	0.0045 (12)	0.0010 (12)	−0.0031 (11)
C10	0.0282 (15)	0.0393 (17)	0.0268 (14)	0.0012 (13)	−0.0028 (12)	−0.0017 (13)
C11	0.0421 (18)	0.0303 (17)	0.0443 (19)	0.0005 (14)	−0.0153 (15)	0.0051 (14)
C12	0.051 (2)	0.040 (2)	0.048 (2)	−0.0110 (16)	−0.0235 (17)	0.0172 (16)
C13	0.060 (2)	0.072 (3)	0.0299 (17)	−0.024 (2)	−0.0086 (16)	0.0155 (18)
C14	0.0404 (18)	0.065 (3)	0.0262 (16)	−0.0016 (17)	−0.0010 (13)	−0.0030 (15)
C15	0.0296 (15)	0.0433 (19)	0.0275 (15)	0.0027 (13)	−0.0014 (12)	0.0001 (13)
Cl1	0.0348 (4)	0.0335 (4)	0.0292 (3)	−0.0071 (3)	−0.0013 (3)	−0.0006 (3)
N1	0.0274 (12)	0.0244 (12)	0.0241 (11)	0.0033 (9)	−0.0002 (9)	−0.0026 (9)
N2	0.0240 (11)	0.0299 (13)	0.0242 (12)	−0.0014 (10)	−0.0028 (9)	0.0022 (10)
O1	0.0349 (11)	0.0493 (14)	0.0251 (10)	−0.0115 (10)	−0.0044 (9)	0.0061 (10)
O2	0.0350 (12)	0.0585 (16)	0.0350 (12)	−0.0179 (11)	−0.0031 (9)	0.0032 (11)
Pt1	0.02348 (6)	0.02556 (6)	0.02184 (6)	0.00129 (4)	−0.00148 (4)	−0.00049 (4)

### Geometric parameters (Å, º)

**Table:**

C1—C2	1.402 (4)	C11—H11B	0.9900
C1—C10	1.507 (4)	C11—C12	1.524 (4)
C1—N1	1.336 (4)	C11—N2	1.486 (4)
C2—H2	0.9500	C12—H12A	0.9900
C2—C3	1.365 (4)	C12—H12B	0.9900
C3—H3	0.9500	C12—C13	1.507 (6)
C3—C4	1.396 (5)	C13—H13A	0.9900
C4—C5	1.419 (4)	C13—H13B	0.9900
C4—C9	1.432 (4)	C13—C14	1.522 (5)
C5—H5	0.9500	C14—H14A	0.9900
C5—C6	1.355 (5)	C14—H14B	0.9900
C6—H6	0.9500	C14—C15	1.526 (4)
C6—C7	1.403 (5)	C15—H15A	0.9900
C7—H7	0.9500	C15—H15B	0.9900
C7—C8	1.369 (5)	C15—N2	1.497 (4)
C8—H8	0.9500	Cl1—Pt1	2.3035 (7)
C8—C9	1.407 (5)	N1—Pt1	2.085 (2)
C9—N1	1.382 (4)	N2—H2A	0.9300
C10—O1	1.295 (3)	N2—Pt1	2.043 (2)
C10—O2	1.217 (4)	O1—Pt1	1.999 (2)
C11—H11A	0.9900		
			
C2—C1—C10	118.8 (3)	H12A—C12—H12B	107.9
N1—C1—C2	123.8 (3)	C13—C12—C11	111.8 (3)
N1—C1—C10	117.4 (2)	C13—C12—H12A	109.3
C1—C2—H2	120.4	C13—C12—H12B	109.3
C3—C2—C1	119.1 (3)	C12—C13—H13A	109.5
C3—C2—H2	120.4	C12—C13—H13B	109.5
C2—C3—H3	120.3	C12—C13—C14	110.8 (3)
C2—C3—C4	119.3 (3)	H13A—C13—H13B	108.1
C4—C3—H3	120.3	C14—C13—H13A	109.5
C3—C4—C5	121.6 (3)	C14—C13—H13B	109.5
C3—C4—C9	119.5 (3)	C13—C14—H14A	109.5
C5—C4—C9	118.9 (3)	C13—C14—H14B	109.5
C4—C5—H5	119.5	C13—C14—C15	110.6 (3)
C6—C5—C4	120.9 (3)	H14A—C14—H14B	108.1
C6—C5—H5	119.5	C15—C14—H14A	109.5
C5—C6—H6	120.1	C15—C14—H14B	109.5
C5—C6—C7	119.8 (3)	C14—C15—H15A	109.4
C7—C6—H6	120.1	C14—C15—H15B	109.4
C6—C7—H7	119.2	H15A—C15—H15B	108.0
C8—C7—C6	121.6 (3)	N2—C15—C14	111.4 (3)
C8—C7—H7	119.2	N2—C15—H15A	109.4
C7—C8—H8	120.0	N2—C15—H15B	109.4
C7—C8—C9	120.1 (3)	C1—N1—C9	118.3 (2)
C9—C8—H8	120.0	C1—N1—Pt1	110.10 (18)
C8—C9—C4	118.7 (3)	C9—N1—Pt1	131.61 (19)
N1—C9—C4	119.9 (3)	C11—N2—C15	110.5 (2)
N1—C9—C8	121.4 (3)	C11—N2—H2A	107.4
O1—C10—C1	114.8 (3)	C11—N2—Pt1	111.61 (18)
O2—C10—C1	120.5 (3)	C15—N2—H2A	107.4
O2—C10—O1	124.7 (3)	C15—N2—Pt1	112.37 (17)
H11A—C11—H11B	107.9	Pt1—N2—H2A	107.4
C12—C11—H11A	109.2	C10—O1—Pt1	115.95 (19)
C12—C11—H11B	109.2	N1—Pt1—Cl1	106.11 (7)
N2—C11—H11A	109.2	N2—Pt1—Cl1	84.26 (7)
N2—C11—H11B	109.2	N2—Pt1—N1	169.63 (9)
N2—C11—C12	111.9 (3)	O1—Pt1—Cl1	171.99 (6)
C11—C12—H12A	109.3	O1—Pt1—N1	81.38 (9)
C11—C12—H12B	109.3	O1—Pt1—N2	88.26 (9)
			
C1—C2—C3—C4	0.8 (5)	C9—N1—Pt1—Cl1	8.2 (3)
C1—C10—O1—Pt1	5.1 (3)	C9—N1—Pt1—N2	−171.6 (4)
C1—N1—Pt1—Cl1	−171.51 (17)	C9—N1—Pt1—O1	−174.7 (3)
C1—N1—Pt1—N2	8.6 (6)	C10—C1—C2—C3	−177.6 (3)
C1—N1—Pt1—O1	5.58 (18)	C10—C1—N1—C9	175.6 (2)
C2—C1—C10—O1	177.8 (3)	C10—C1—N1—Pt1	−4.7 (3)
C2—C1—C10—O2	−0.7 (4)	C10—O1—Pt1—N1	−6.0 (2)
C2—C1—N1—C9	−2.1 (4)	C10—O1—Pt1—N2	174.6 (2)
C2—C1—N1—Pt1	177.6 (2)	C11—C12—C13—C14	−53.6 (4)
C2—C3—C4—C5	178.4 (3)	C11—N2—Pt1—Cl1	−129.0 (2)
C2—C3—C4—C9	0.4 (4)	C11—N2—Pt1—N1	50.9 (6)
C3—C4—C5—C6	−176.6 (3)	C11—N2—Pt1—O1	53.9 (2)
C3—C4—C9—C8	175.7 (3)	C12—C11—N2—C15	−56.3 (3)
C3—C4—C9—N1	−2.5 (4)	C12—C11—N2—Pt1	177.9 (2)
C4—C5—C6—C7	0.3 (6)	C12—C13—C14—C15	54.5 (4)
C4—C9—N1—C1	3.3 (4)	C13—C14—C15—N2	−56.9 (4)
C4—C9—N1—Pt1	−176.4 (2)	C14—C15—N2—C11	57.6 (3)
C5—C4—C9—C8	−2.3 (4)	C14—C15—N2—Pt1	−177.0 (2)
C5—C4—C9—N1	179.4 (3)	C15—N2—Pt1—Cl1	106.25 (19)
C5—C6—C7—C8	−1.3 (6)	C15—N2—Pt1—N1	−73.9 (6)
C6—C7—C8—C9	0.3 (6)	C15—N2—Pt1—O1	−70.9 (2)
C7—C8—C9—C4	1.5 (5)	N1—C1—C2—C3	0.0 (5)
C7—C8—C9—N1	179.7 (3)	N1—C1—C10—O1	0.0 (4)
C8—C9—N1—C1	−174.9 (3)	N1—C1—C10—O2	−178.5 (3)
C8—C9—N1—Pt1	5.4 (4)	N2—C11—C12—C13	54.9 (4)
C9—C4—C5—C6	1.4 (5)	O2—C10—O1—Pt1	−176.5 (3)

### Hydrogen-bond geometry (Å, º)

**Table:**

*D*—H···*A*	*D*—H	H···*A*	*D*···*A*	*D*—H···*A*
N2—H2*A*···Cl1^i^	0.93	2.74	3.624 (2)	160
C3—H3···O2^ii^	0.96	2.53	3.360 (4)	145
C8—H8···Cl1	0.95	2.40	3.268 (3)	152

Symmetry codes: (i) −*x*+1, *y*, −*z*+3/2; (ii) *x*, −*y*+1, *z*−1/2.
